# Effect of selenium nano-vaccine on hematological biomarkers and immune biochemical activity of nile tilapia (*Oreochromis niloticus*) challenged with *Streptococcus pyogenes*

**DOI:** 10.1038/s41598-025-27004-x

**Published:** 2025-11-21

**Authors:** Sameh Nasr-Eldahan, Asmaa Nabil-Adam, Mohamed Attia Shreadah, Adham M. Maher, Tamer El-Sayed Ali

**Affiliations:** 1 Oceanography Department, Faculty of Science, Alexandria University, Alexandria, Egypt; 2 National Institute of Oceanography and Fisheries (NIOF), Alexandria, Egypt; 3 Biochemistry Department, Faculty of Science, Alexandria University, Alexandria, Egypt

**Keywords:** Selenium nanoparticles, Vaccine, Liver, Streptococcus pyogenes, Nile tilapia, Biochemistry, Biotechnology

## Abstract

*Streptococcus pyogenes* infection in Nile tilapia causes high mortality and economic loss. This study evaluated the efficacy of a formalin-killed *S. pyogenes* bacterium loaded on nano-selenium (nano-vaccination) to prevent streptococcal disease outbreaks on farmed tilapia. 120 Nile tilapia fingerlings were allocated into 4 groups: control group: was injected with 0.1mL of normal saline without any challenges, infected group: was injected with 0.1mL of 1 × 10^7^ (CFU)mL^− 1^, nano-vaccine group: was injected with two doses of nano-vaccine with an interval of 21 days until day 32nd and nano-vaccine bacteria group: was injected with two doses of nano-vaccine then challenge with bacteria after day 32nd. Hematological biomarkers, inflammatory, oxidative stress, antioxidant enzymes, and histology were estimated in the liver. The selenium nano-vaccinated group showed significant improvement in hematological profiles and elevated antioxidant enzyme activity compared with the control. Compared to the unvaccinated fish, the vaccinated fish showed an enhancement in antioxidant capacity and hematological stability after bacterial challenge. In contrast, infected fish showed higher oxidative stress and inflammatory indicators. Selenium nano-vaccination enhanced hematological performance, antioxidant defense, and immune response in Nile tilapia, allowing effective protection against S. pyogenes infection with minimal inflammation and oxidative damage.

## Introduction

Like other aquatic species, tilapias are subjected to several diseases and parasites. Fish have both specialized and non-specialized disease defenses. The Nile tilapia (*Oreochromis niloticus*) is more susceptible to disease than many other freshwater species, while being one of the most promising fish in the aquaculture industry^1^. During the second half of the twentieth century, there were outbreaks in tropical, subtropical, and temperate areas, one of the causes of tilapia infection in *Streptococcus* species^2^. Infection by *Streptococcus* species has been reported in tilapia farms, causing Streptococcosis diseases^3^. Approximately 60% of all infected individuals died from *Streptococcus pyogenes*, which is considered one of the most prevalent Streptococcus species^4^. *Streptococcus pyogenes* is a type A *streptococcus* that causes streptococcosis. In addition to additional signs, including external multi-focal hemorrhages around the mouth or at the base of the fins, this condition causes fish to become disoriented, sluggish, and twisted in their bodies^5,6^.

Diverse fish species have been shown to possess unique defense mechanisms, such as distinct immunological responses to infections^7^. And to enhance this immune response, the vaccine is widely used in aquaculture for this purpose, especially in farmed salmon and the koi herpes virus^8^. Animals’ immune systems are stimulated by vaccines to become more resilient to diseases brought on by common infections in the future. B lymphocytes, which produce antibodies in response to vaccine stimuli, stay sensitive and prepared to react to the agent should it ever enter the body^9^. Traditional vaccines are formed by killing the infectious agent with formalin and using it as an antigen to stimulate the immune system^10^. But with the introduction of nanotechnology in vaccine formation, the vaccine will be more effective. It has been considered a solution that has a beneficial role in preventing and monitoring diseases and pathogens in the aquaculture industry^11^.

Immunizations made with an antigen or set of antigens that contain the appropriate nanoparticle are known as nano-vaccines. They are a novel family of vaccines that precisely target the infection site through immune system targeting, thereby stopping the spread of infections and illnesses^12^. Make use of nanoparticles as carriers and/or adjuvants to boost vaccination immunogenicity. Due to viruses’ and nanoparticles’ comparable sizes, the immune system can be successfully stimulated, resulting in the induction of cellular and humoral immunity responses^6^. The principal benefit of the nano-vaccine is the enhancement of fish immunity. where the highly heterologous cells that are involved in innate immunity, such as monocytes and macrophages, are extensively disseminated. According to^13^ macrophages present and process antigens to elicit an adaptive immune response. Macrophages are readily targeted by surface-engineered nanoparticles because of their innate phagocytic nature and their capacity to combine cognate ligands that bind to macrophage receptors. To promote interactions between nanoparticles and macrophage receptors, a variety of physicochemical properties of NPs, including thickness, surface load, hydrophobicity, surface topography, and material structure, can be improved. Not to mention, T and B cells in the adaptive immune system have a variety of receptors that allow them to recognize different antigens. The T-cell immune system’s ability to activate or suppress an illness may determine its outcome. Many treatment strategies based on nanoparticles have been developed to control T-cell activity against bacterial, fungal, or viral infections^14,15^, showing that B cells may recognize and react to microbial surface antigens through B-cell receptors. To produce vaccinations for a variety of diseases, engineered nanoparticles were utilized to clonally multiply and activate antigen-specific B-cells^6^.

The objective of this study, therefore, is to investigate the potential of selenium nano-vaccine in protecting Nile tilapia (*Oreochromis niloticus*) fingerlings from microbial infections induced by *Streptococcus pyogenes*. This was accomplished by enhancing fish’s natural immunity and lowering the infectious disease virulence, because of raising the total antioxidant enzymes and thus reducing the oxidative stress.

## Materials and methods

### Fish acclimatization

One hundred and twenty-one healthy Nile tilapia (*Oreochromis niloticus*) fingerlings were netted from Elmadina Hatchery, Kafr-Elshekish Governorate, Egypt. Furthermore, transported in a plastic container supplied with oxygen and contained pond water to the Marine Biotechnology and Natural Products Lab (MBNP) research animal unit at the National Institute of Oceanography and Fisheries (NIOF), Alexandria, Egypt. Upon transportation for three weeks, all fish were kept in 1000-liter plastic tanks that were continuously aerated and supplied with dechlorinated tap water to check the fish’s health state and ensure it was free from any disease symptoms. All fish received commercial pellets (30% crude protein, El-Ekhwa factory, Egypt) twice daily at 8:00 a.m. and 02:00 p.m. within 4% of the total biomass.

### Experimental design

After acclimatization, Nile tilapia (120 specimens; 15 ± 0.1 g/fish) were carefully transported to a glass aquarium (60 L) with well-aerated dechlorinated tap water. The water temperature was monitored using thermometers (50-watt Chxilong^®^ heater) and kept constant at 27 ± 1 °C. pH (7.5-8), ammonia (0.0016 ppm), and dissolved oxygen (7–9 mg/l) were maintained at previous levels by exchanging 25% of the water every day (Ayoub, 2021). The fish divided into four groups in triplicate as shown in Tabel 1, control groups: injected with 0.1 ml of normal saline without any challenges, infected group: injected with 0.1 ml of 1 × 10^7^ (CFU) ml^− 1^ of *Streptococcus pyogen*, selenium nano-vaccine group (Se-NV): injected with two doses of 0.1 ml/100 g fish body weight with an interval of 21 days until day 32nd, and finally, selenium nano-vaccine group (Se-NV + inf): in day 32nd after nano vaccine injection, *Streptococcus pyogen* challenge done by inject fish with 0.1 ml of 1 × 10^7^ (CFU) ml^− 1^^10^.

**Table 1:** Distribution of tested fish species among the groups.

Treatment	Description	Injection dose
1	Control	0.1 ml of normal saline
2	Infected with bacteria	0.1 ml of 1 × 10^7^ (CFU) ml^− 1^
3	Se-NV	0.1 ml/100 g
4	Se-NV + inf	0.1 ml/100 g + 0.1 ml of 1 × 10^7^ (CFU) ml^− 1^

### Experimental termination

At the end of the experiment, all fish were fasted for 24 h, then anesthetized using tricaine methane sulfonate (MS-222) (50 mg/L)^16^. Then blood was extracted from the caudal region of each fish and then was put into a sodium EDTA tube. It is then promptly analyzed using a hematological device to determine its current condition, such as white blood cells (WBCs), lymphocytes (LYM), red blood cells (RBCs), and hemoglobin (HGB), etc. According to^17^, the fish’s liver was extracted. where three cuts are done. The first cut started from the anal opening and guided through the central part of the abdominal wall toward the gills. The second cut began from the anal opening and was directed parallel to the lateral line, finishing at the gill’s dorsal end. The third cut links the first and second cut ends. After that, the tissue was homogenized using potassium phosphate buffer to calculate the activities of Catalase (CAT), Superoxide dismutase (SOD), Glutathione (GSH), Glutathione-S-Transferase (GST), Glutathione reductase (GR), and Glutathione peroxidase (GPx) following the methods described by^18–23^ respectively. Malondialdehyde (MDA), a lipid peroxidation marker, was examined using the description methods by^24^. Finally, for liver histopathology, liver tissue specimens were taken from all individual groups according to the method described by^25^.

### Selenium nano vaccination preparation and evaluation

#### Bacterial culture and inactivation

*Streptococcus pyogenes* was obtained from the American Type Culture Collection (ATCC), Virginia, United States. Then cultured in the nutrient broth media (Trypticase Soy Agar with 5% sheep blood), according to^26^, and inactivation by adding 10% formalin according to^27^.

#### Preparation of Se-NPs using marine polymer “Chitosan”

Selenium nanoparticles (Se-NPs) were formed using green synthesis by reducing sodium selenite with chitosan as a marine polymer, and vitamin C, according to^28^. Briefly, 0.5 g of chitosan (CTS) and 1.6 g of vitamin C were dissolved in 100 mL of 1% (w/w) acetic acid. Then, add selenite aqueous solution (dissolved 0.4 g of sodium selenite in 10 ml of deionized water) slowly with vigorous stirring (600–800 rpm) to obtain a CTS-Se-NPs colloid. Then dialyzed against 1% (w/w) acetic acid for 6 h to remove excess vitamin C and other by-products.

#### Loading formalin-killed bacteria on Se-NPs (selenium nano-vaccination)

Selenium nano-vaccine was prepared according to^29^. Firstly, the fresh Se-NPs were prepared. Secondly, *Streptococcus pyogenes* formalin-killed was prepared. Then added inactivated *Streptococcus pyogenes* was added to Se-NPs (size < 100 nm) at a concentration of 0.3% mg/Kg and was incubated overnight at 4 °C with gentle stirring.

#### Selenium nano-vaccine safety evaluation

Fish were injected intraperitoneally (i.p.) after being anesthetized with 0.1 ml of vaccine (ten fish per group) and kept for two weeks. After injection, the fish were examined daily for symptomatic adverse effects such as behavioral changes, lesions around the injection site, and immediate mortality rates due to toxicity^30^.

### Statistical analysis

Data was analyzed statistically using the IBM SPSS software package version 20.0. (Armonk, NY: IBM Corp). Qualitative data were described using numbers and percentages. The Shapiro-Wilk test was used to verify the normality of the distribution. Quantitative data were described using range (minimum and maximum), mean, and standard deviation. Significance of the results obtained was judged at the 5% level. The F-test (ANOVA) for normally distributed quantitative variables was applied to compare more than two groups, and the Post Hoc test (Tukey) for pairwise comparisons.

##  Results

### Safety assessment of vaccines

The first step in determining the safety of selenium nano-vaccine formulations involved injecting them intravenously into fish and monitoring the animals for a period of two weeks. After injectable inoculations, no mortalities were recorded in different groups, and during the two weeks that followed the injection, no unusual behavior was observed. Additionally, all fish groups maintained their health throughout the large inoculation experiment, and no mortalities, acute toxicity indicators, or long-term negative effects were noted.

### Hematological biomarkers

**Fig. 1:**
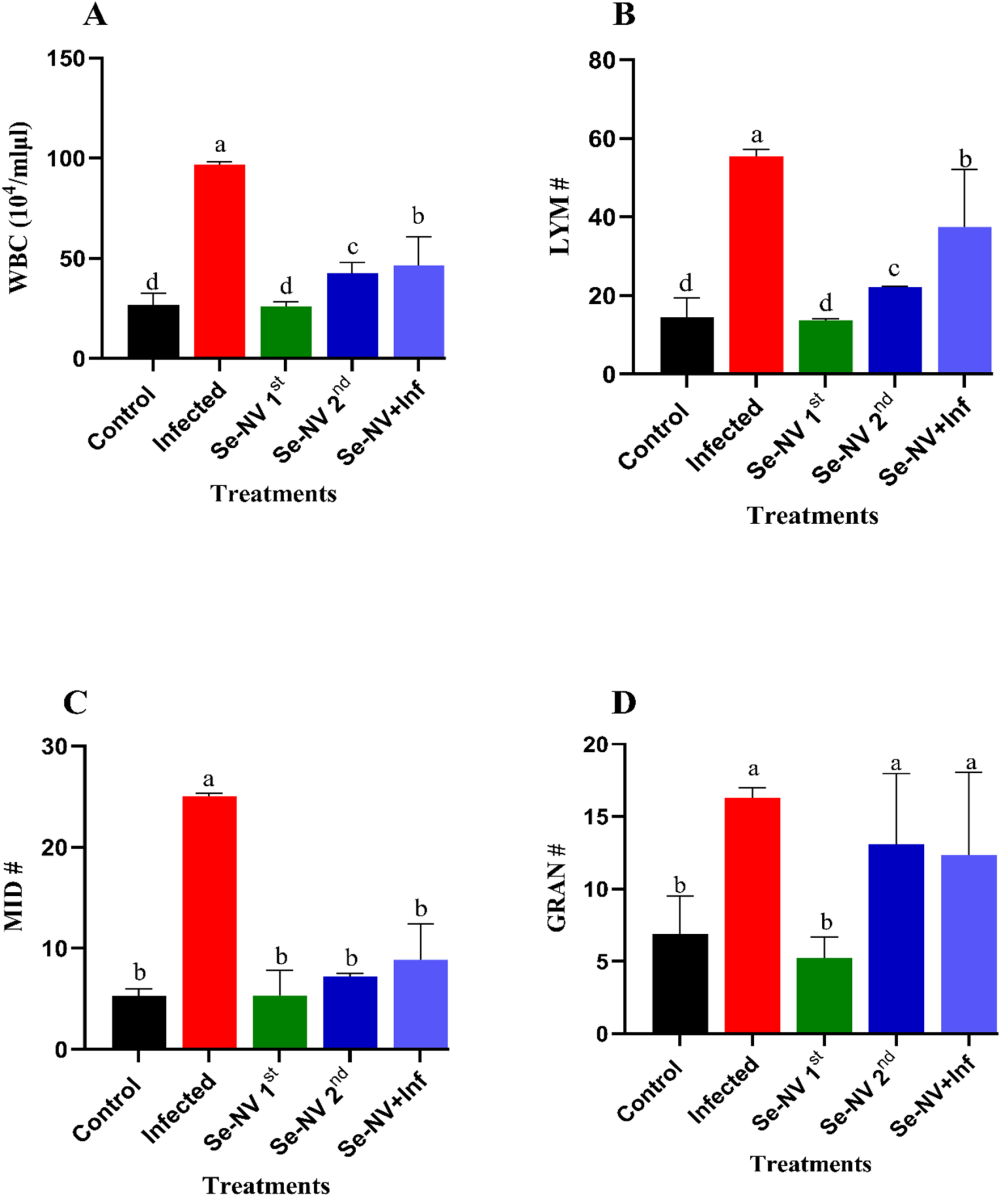
Concentration of (**A**) white blood cell “WBC”, (**B**) lymphocytes “LYM”, (**C**) Mixed Cells Count “MID”, and (**D**) granulocyte (GRAN) under different treatments. Data are presented as mean ± standard deviation. Treatments: control (injection with saline), infected (injection with bacteria), Se-NV 1st (first vaccine injection), Se-NV 2nd (Second vaccine injection), and Se-NV + Inf (challenge with bacteria after vaccine injection).

Figure 1 shows the hematological profile and its components. WBC (Fig. 1A) showed a significant increase in the bacterial group (infected) compared with the control (96.77 ± 1.51 vs. 26.7 ± 5.87). Also, 1st and 2nd injections of the vaccinated (Se-NV) group showed a significant decrease compared with the infected group, with a significant increase in 2nd compared with 1st (42.4 ± 5.37 vs. 25.77 ± 2.65). Furthermore, the vaccine bacteria group (Se-NV + Inf) showed a significant decrease compared with the infection group (46.37 ± 14.28 vs. 96.77 ± 1.51), with a significant increase compared with the control. In LYM # (Fig. 1B), Se-NV 1st and 2nd injections of the Se-NV group showed a significant increase compared with the control group (13.63 ± 0.5 & 22.1 ± 0.17 vs. 14.53 ± 4.88), with a significant increase in 2nd compared with 1st. Also, the infection group showed the highest value compared with other groups. In Se-NV + Inf, there was a significant decrease compared with the infection group (37.40 ± 14.74 vs. 55.43 ± 1.78) and a significant increase compared with Se-NV. In MID # (Fig. 1C), the highest value was shown in the infected group (25.06 ± 0.25). Furthermore, there were no significant differences among the Se-NV 1st, 2^nd,^ and control groups (5.33 ± 2.48 vs. 7.23 ± 0.29 vs. 5.30 ± 0.72). In contrast, Se-NV + Inf showed a significant decrease compared with the infected group (8.87 ± 3.58 vs. 25.06 ± 0.25) and showed no significant differences with the control group. Finally, in GRAN# (Fig. 1D), the highest level was shown in the infected, Se-NV 2^nd,^ and Se-NV + Inf groups (16.27 ± 0.71 vs. 13.07 ± 4.91 &12.33 ± 5.71) compared with the control group (6.87 ± 2.66), but there was no significant difference between Se-NV 1st and control.

**Fig. 2:**
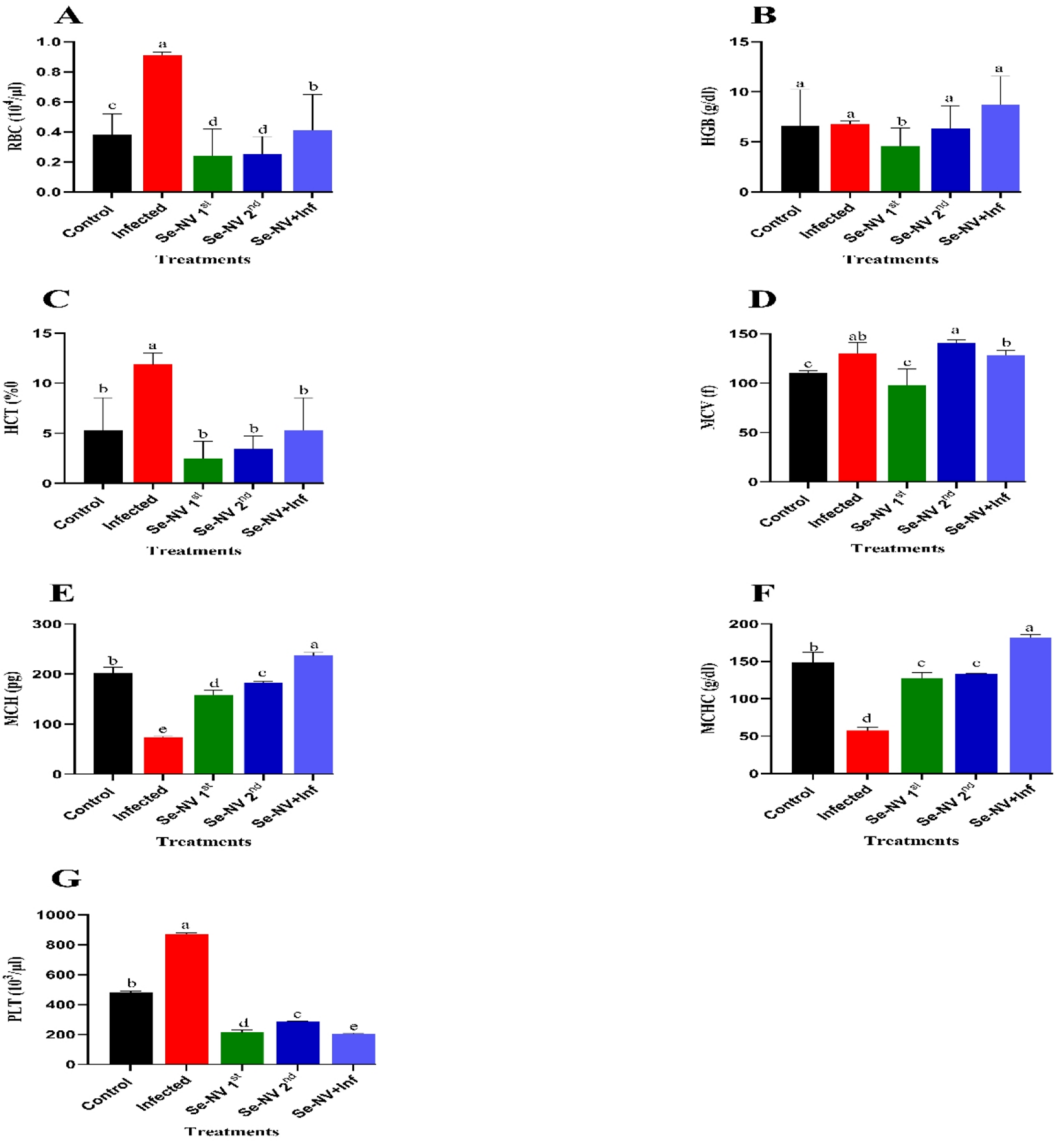
Concentration of (**A**) red blood cell “RBC”, (**B**) hemoglobin “HGB”, (**C**) hematocrit “HCT”, (**D**) Mean Corpuscular Volume “MCV’, (**E**) Mean Corpuscular Hemoglobin “MCH’, (**F**) Mean Corpuscular Hemoglobin Concentration “MCHC”, and (**G**) platelet (PLT) under different treatments. Data are presented as mean ± standard deviation. Treatments: control (injection with saline), infected (injection with bacteria), Se-NV 1st (first vaccine injection), Se-NV 2nd (Second vaccine injection), and Se-NV + Inf (challenge with bacteria after vaccine injection).

RBC’s profile and its components are represented in Fig. 2. RBCs (Fig. 2A) showed the highest value in the infection group (0.91 ± 0.02). With Se-NV 1st and 2^nd,^ there was no significant difference, but showed a significant decrease compared with control (0.24 ± 0.18 and 0.25 ± 0.12 vs. 0.38 ± 0.14). Also, Se-NV + Inf showed no significant differences with control (0.41 ± 0.24 vs. 0.38 ± 0.14). In HGB (Fig. 2B), there were no significant differences between Se-NV 2nd, infected, Se-NV + Inf, and control, with a significant increase compared with Se-NV 1st (6.37 ± 2.25 & 6.77 ± 0.29 & 6.63 ± 3.59& 8.73 ± 2.81 vs. 4.6 ± 1.79). In the HCT level (Fig. 2C), the highest values were shown in the infected group (11.87 ± 1.14). In contrast to Se-NV 1st, 2nd, control, and Se-NV + Inf, which showed the lowest values without any significant differences among them (2.5 ± 1.67 vs. 3.43 ± 1.31vs 5.27 ± 3.29 vs. 5.27 ± 3.29). MCV (Fig. 2D) showed no significant differences between infection and Se-NV + Inf groups (130.4 ± 10.74 vs. 128 ± 5.44). Also, there were no significant differences between infected and Se-NV 2nd (130.4 ± 10.74 vs. 141 ± 2.83), which showed the highest values. In contrast, the low values were shown in Se-NV 1st and control (97.97 ± 16.76 vs. 110.3 ± 2.37) without any significant differences. With MCH level (Fig. 2E), there was a significant increase in Se-NV 2nd compared with Se-NV 1st (183.5 ± 1.44 vs. 158.2 ± 9.21); in contrast, MCHC level (Fig. 2F) showed no significant differences between them (133.8 ± 0.4 vs. 127.3 ± 7.76). Also, the lowest values for MCH & MCHC were shown in the infected groups compared with the control (74.03 ± 1.27 &57.2 ± 4.39 vs. 202 ± 11.3& 148.5 ± 13.4), in contrast to Se-NV + Inf, which showed the highest values in MCH & MCHC compared with the control (236.7 ± 6.05&182 ± 3.89 vs. 202 ± 11.3& 148.5 ± 13.4). The PLT (Fig. 2G) showed the highest value in the infected group compared with the control (873.3 ± 4.61 vs. 483 ± 5.58), also Se-NV 2nd showed a significant increase compared with Se-NV 1st (287.3 ± 3.66 vs. 213 ± 17.8). In Se-NV + Inf, which showed the lowest value, there was a significant decrease compared with control (206 ± 3.54 vs. 483 ± 5.58).

### Inflammatory markers

**Fig. 3:**
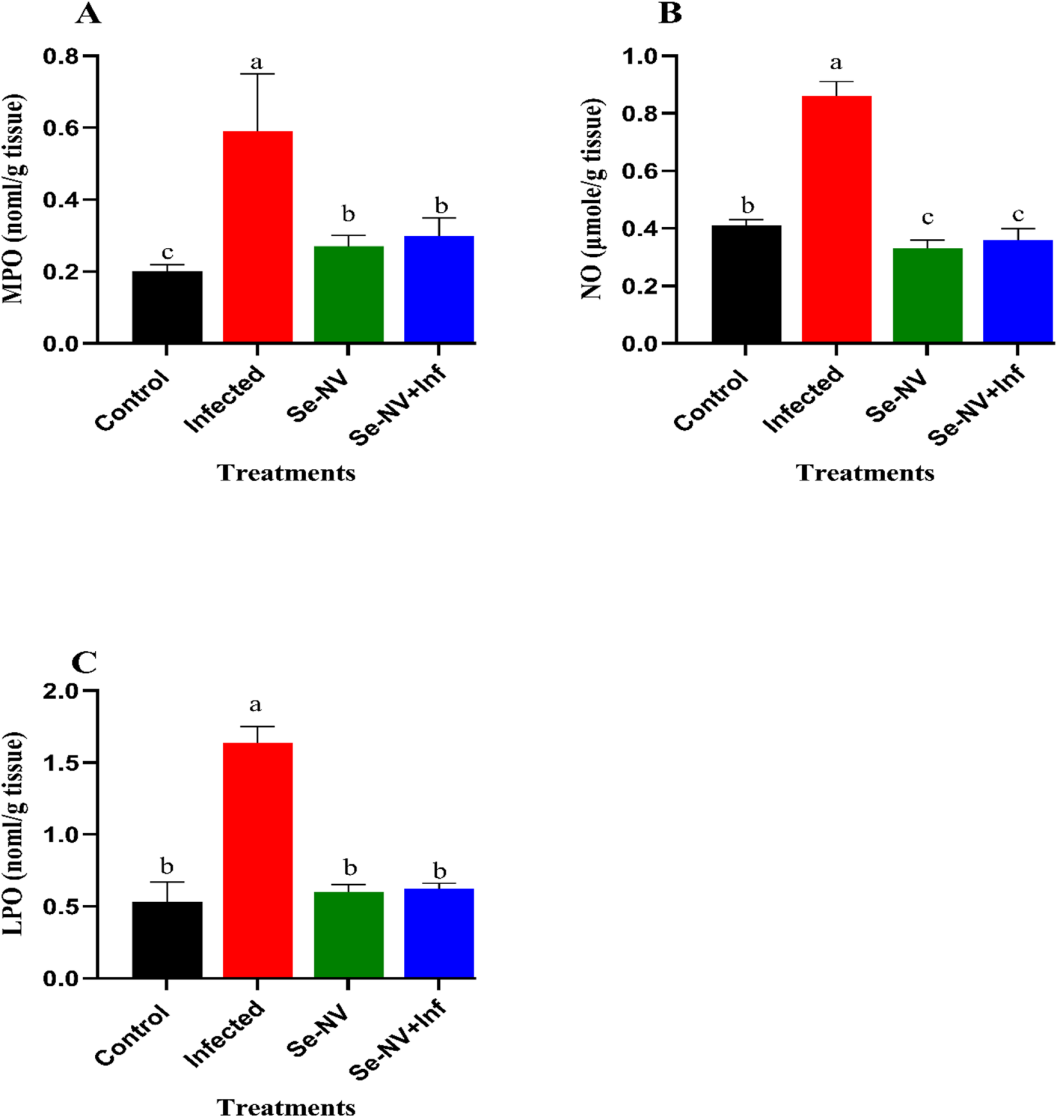
Concentration of (**A**) myeloperoxidase “MPO”, (**B**) nitric oxide “NO”, and (**C**) lipid peroxidation “LPO” under different treatments. Data are presented as mean ± standard deviation. Treatments: control (injection with saline), infected (injection with bacteria), Se-NV (vaccine injection), and Se-NV + Inf (challenge with bacteria after vaccine injection).

Figure 3 depicts the levels of inflammatory myeloperoxidase (MPO), nitric oxide (NO), and lipid peroxidation (LPO) in the liver of the vaccine-injected groups before and after the bacterial challenge. The determination of MPO levels (Fig. 3A) revealed a significant increase in the liver-infected group compared with the control group (0.59 ± 0.16 vs. 0.2 ± 0.02). In the case of Se-NV and Se-NV + Inf, a significant decrease in MPO levels was shown (0.27 ± 0.03 & 0.3 ± 0.05) compared with the Infected group, without any significant difference between Se-NV and Se-NV + Inf groups. Furthermore, NO levels (Fig. 3B) showed the same results with a significant increase in infected groups compared with control (0.86 ± 0.05vs0.41 ± 0.02), and significant decreases in Se-NV and Se-NV + Inf (0.33 ± 0.03 & 0.36 ± 0.04). Finally, LPO levels (Fig. 3C) showed the highest value was shown in the infected group compared with the control (1.64 ± 0.11 vs. 0.53 ± 0.14). Furthermore, the lowest values were shown in Se-NV and Se-NV + Inf (0.60 ± 0.05 & 0.62 ± 0.04), respectively. These previous results showed the highest values in the infection group, in contrast to Se-NV and Se-NV + Inf.

### Antioxidant enzyme statuses

**Fig. 4:**
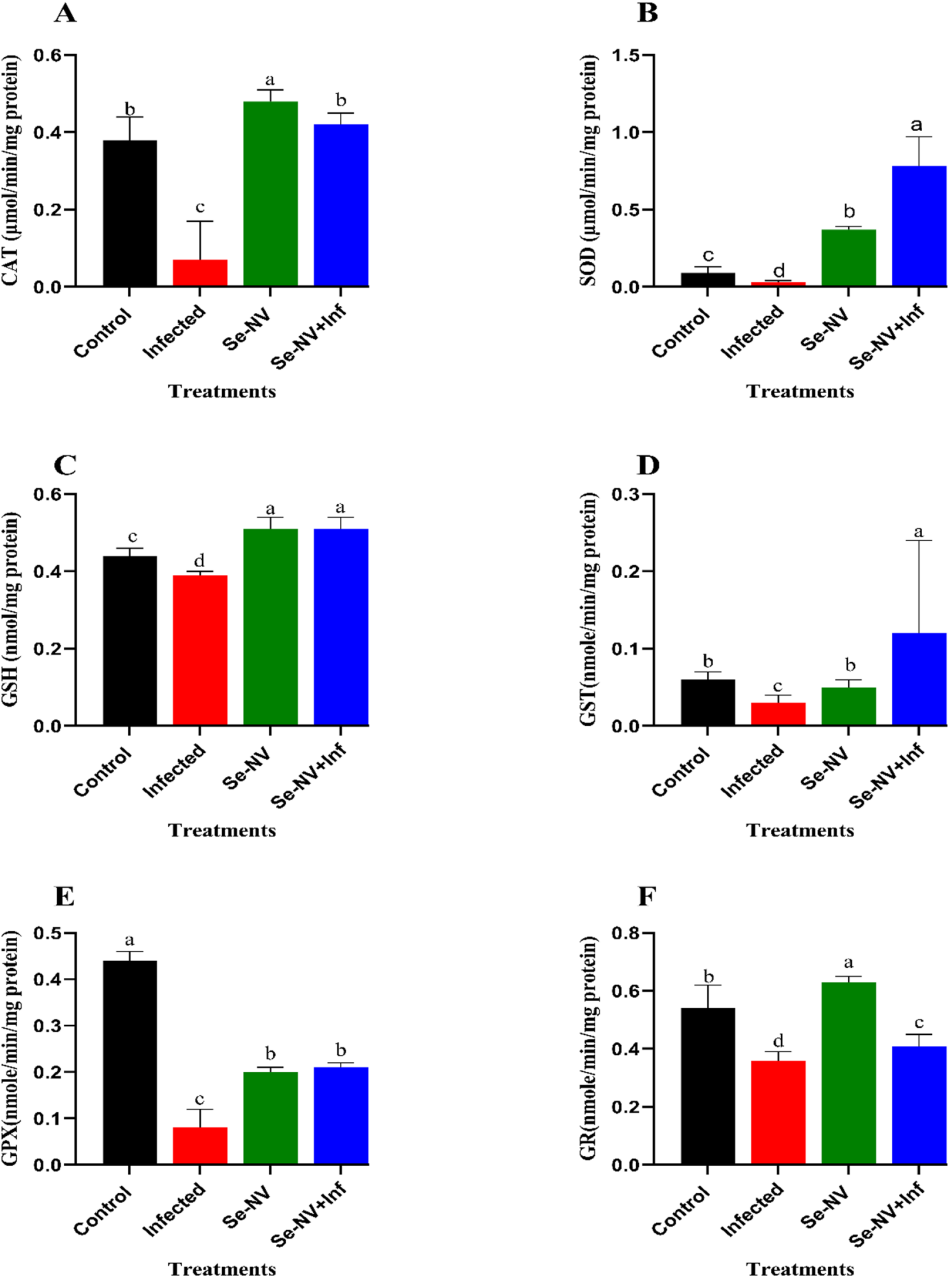
Concentration of antioxidant enzymes, (**A**) catalase “CAT”, (**B**) superoxide dismutase “SOD”, (**C**) Glutathione “GSH”, (**D**) Glutathione S-transferase ‘GST’, (**E**) Glutathione peroxidase “GPX”, and (**F**) glutathione reductase “GR” under different treatments. Data are presented as mean ± standard deviation. Treatments: control (injection with saline), infected (injection with bacteria), Se-NV (vaccine injection), and Se-NV + Inf (challenge with bacteria after vaccine injection).

Catalase (CAT), superoxide dismutase (SOD), reduced glutathione (GSH), glutathione-S-transferase (GST), glutathione peroxidase (GPx), and glutathione reductase (GR) were estimated in the vaccinated liver before and after challenge with bacteria Fig. 4. In CAT levels (Fig. 4A) there was a significant increase in Se-NV and Se-NV + Inf groups compared with the control groups (0.48 ± 0.03 & 0.42 ± 0.03 vs. 0.38 ± 0.06); in contrast infected group showed a significant decrease (0.07 ± 0.01) compared with the control group. Furthermore, the same results showed in SOD levels (Fig. 4B), with a significant increase in Se-NV and Se-NV + Inf groups and a significant decrease in the infected group compared with the control (0.37 ± 0.02 & 0.78 ± 0.19&0.03 ± 0.01 vs. 0.09 ± 0.04). In GSH (Fig. 4C), there was a significant increase in Se-NV and Se-NV + Inf groups compared with the control group (0.51 ± 0.03 & 0.51 ± 0.03 VS 0.44 ± 0.02); in contrast infected group showed a significant decrease (0.39 ± 0.01) compared with the control group. Furthermore, the same results were shown in GST (Fig. 4D), with values of Se-NV, Se-NV + Inf, infection, and control groups (0.05 ± 0.01&0.12 ± 0.12 &0.03 ± 0.01 vs. 0.06 ± 0.01), respectively. Finally, GPX levels (Fig. 4E) showed a significant decrease in Se-NV and Se-NV + Inf groups compared with control groups(0.20 ± 0.01&0.21 ± 0.01vs0.44 ± 0.02). Also, the infected group showed a significant decrease (0.08 ± 0.04) compared with the control group. Furthermore, the same results showed in GR levels (Fig. 4F) with a significant increase in Se-NV and Se-NV + Inf and a significant decrease in infected group values compared with the control (0.63 ± 0.02&0.41 ± 0.04 &0.36 ± 0.03 vs. 0.54 ± 0.08, respectively. These previous results showed the highest values in the Se-NV and Se-NV + Inf groups, in contrast to the infection group.

### Liver histopathological

**Fig. 5:**
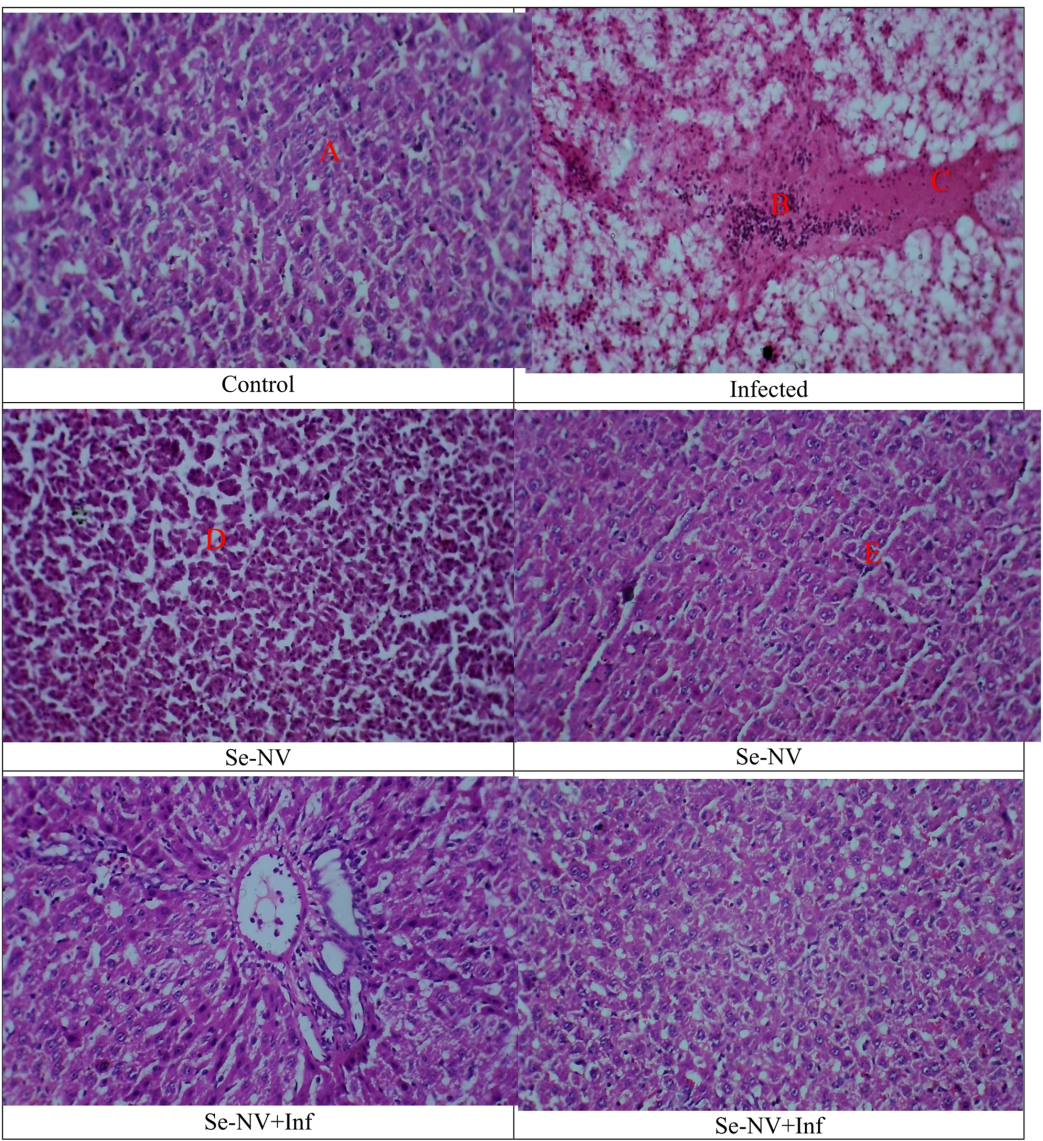
Liver histological changes in different groups. Treatments: control (injection with saline), infected (injection with bacteria), Se-NV (vaccine injection), and Se-NV + Inf (challenge with bacteria after vaccine injection).

Figure 5 shows the different changes in the Tilapia liver. The control group showed hepatocytes in a uniform cord that extends from the central vein. The hepatocytes have a polygonal appearance as they have uniform cytoplasm and numerous nuclei. There are no symptoms of vacuolization, cellular degeneration, necrosis, or inflammatory infiltration demonstrated. Overall, the liver section showed healthy liver morphology. Furthermore, the Se-NV group has the same results, which showed liver in normal morphology without any notable necrosis, inflammation, steatosis, or fibrosis. In contrast, the infected group showed a change in liver morphology, including hepatocellular degeneration and vacuolization in the hepatic tissue (Fig. 5A). Furthermore, there was a breakdown in cytoplasm and lipid accumulation, which is considered a sign of fatty degeneration. Also, an inflammatory response was demonstrated by the presence of inflammatory cells within the hepatic cells surrounding the central vein (Fig. 5B). Overall, these findings indicate hepatic stress and decreased metabolic function, which may be caused by oxidative stress due to bacterial infection. Finally, in Se-NV + Inf, an improvement is shown in the arrangement of hepatocytes (Fig. 5C). in the liver, with little vacuolation and necrosis (Fig. 5D).

##  Discussion

Hematological characteristics are a valuable tool that can be used as an effective and sensitive indicator for tracking physiological and pathological changes in fish^31^. In the current study were compared hematological parameter among *Streptococcus pyogenes* bacteria (infected group), Selenium nano-vaccine (Se-NV), and Selenium nano-vaccine Bacteria (Se-NV + Inf) groups. The increase of WBCs, lymphocytes, and Neutrophils (a type of granulocyte) in the infected group indicates a response of the body’s resistance to disease-causing antigens, considering a natural reaction to the presence of the bacterial pathogen by induction of the nonspecific defense system^32^. Furthermore, indicated an increase in the body’s immunity, as evidenced by increased activity of phagocyte cells, which act to perform phagocytosis against foreign objects entering the fish body^33^. Furthermore, the increase in neutrophils could be due to the response to a bacterial infection, as neutrophils escape from the marginal group and join the infection area, and the thymus releases its source of reserve, resulting in increased granulopoiesis, with this increase due to the presence of many immature cells. Neutrophils entering the blood circulation, killing, and digesting bacteria^34^, and this agrees with^35^. The elevation of WBCs, lymphocytes, and neutrophils in the Se-NV and Se-NV + Inf groups. This could be due to increased antibody production and/or the movement of leukocytes carrying the nano-vaccine from blood vessels to specific target cells. When the fish body received selenium nano-vaccine WBCs, and other components increased into the injection site, the fish body did not consider the Se-NV to be a harmful agent because it contains Se-NPs, which act as a nano-carrier to the adjuvant. Leukocytes recognize Se-NV after it has been assembled and incubated, and then the leukocytes activate endocytosis or phagocytosis pathways to internalize nanoparticles^36^. Phagocytic cells that express membrane receptors for nanoparticle internalization include monocytes, neutrophils, and macrophages. To increase Se-NV uptake, Fc receptors, scavenger receptors, and mannose receptors have been studied^37^. After the nanoparticle-leukocyte compounds are formed, the nano vaccine and leukocyte move together without any modifications in the effectiveness of the nano vaccine or the functions of the leukocytes into the target or disease site^38^. In the nano-vaccine bacteria group, increasing WBCs, lymphocytes, and Neutrophils count than the Se-NV + Inf group may be because Nile tilapia is giving a response toward the infected bacteria that enter the fish body, resulting in the activation and the movement of the monocyte and macrophage, besides to leukocytes nano-vaccine complex into the specific infected site. This leads to an improvement in the immune response due to more antibody production to destroy or lyse bacteria^39^.

The slight decrease in HGB in the infected group could be due to the deterioration of the haemotopoietic organs located in the spleen and pronephros, where the bacteria may cause pathologies in the haemopoietic organs, particularly in the fish liver, which leads to a decrease in the production of HGB and other hematological parameters^40^. This decrease in these hematological parameters is an indicator of anemia^41^. The same results were obtained when fish were infected with *S. agalactiae* bacteria^42^, also^43^show the same results when catfish were infected with *A*. *hydrophila*, and when Nile tilapia were infected with *Flavobacterium columnare.* Like innate immune cells, platelets (PLT) contain PRRs (pattern recognition receptors), which recognize different components that are increased during infection in the infected group, showing an increase in platelet count after infection. Bacteria release toxins called estreptolysin O, which can activate platelets, increase platelet production, and cause platelet aggregation^44^. Also, during invasive infection, *S.pyogenes* bacteria may use a cloak of fibrinogen and activated platelets to evade the immune response and spread through the bloodstream. *S.pyogenes* bacteria could bind with fibrinogen to increase bacteria’s survival in the bloodstream and bind with fibrinogen^45^. In Se-NV and Se-NV + Inf groups, increasing HGB, MCV, and MCHC may be due to Se-NPs in nano-Vaccine acting as a carrier of the adjuvant, where it is derived adjuvant by an intravenous delivery system into the target tissue. RBCs play a significant role in the circulating environment, as they can transfer NPs or inspire NPs for adjuvant delivery systems that can transport DNA, protein, and adjuvant to target tissues, cells, and organs^46^. So, when fish are injected with Se-NV, the RBCs recognize it, increase production, and carry or absorb the nano-vaccine, improving stream flow^47^. The Se-NPs, which are found in Se-NV, can improve the equilibrium and longevity of RBC membranes. Furthermore, the increase of HGB and MCHC in the nano-vaccine group may be due to absorbed and linked RBC with the nano-vaccine, leading to HGB to be stimulated to increase production^47^. Regardless of the presence of Se-NPs that impedes hemoglobin and iron absorption in the intestine, which causes iron to rise, leading to an increase in hemoglobin, which prevents anemia and ensures good oxygen transmission between cells. Also, increasing MCH and MCHC following an increase in HGB level^48^.

Myeloperoxidase (MPO) and nitric oxide (NO) are considered inflammatory biomarkers and active signaling elements involved in the regulation of various pathophysiological processes in immunological systems^49^. Exogenous polysaccharides, which are antigenic agents, may have been produced in large numbers in the infected group, which is reason NO and MPO increased^50^, which can enhance macrophages to produce large amounts of NO and MPO^51^, may have multiple functions in immunity, resulting in cell damage, and can cause acute or chronic inflammation, and this is agreeing well with^49^.On contract, Se-NV and Se-NV + Inf groups, which show a decreasing may be used to describe how the presence of Se-NPs, which function as a nano carrier for adjuvant, might reduce inflammatory reaction by boosting local immunity, which may improve resilience to infectious diseases and oxidative stress. Because the selenium nano carrier increases endogenous NO synthesis, inhibits NO metabolism, and activates NOS systems, it improves NO bioavailability and has a favorable impact on regulating the distribution and release of NO molecules in the body^52^. In addition to increasing the agent’s availability to the systemic circulation, nano carriers can enhance the interaction between nitric oxide and blood vessels, or the target of NO, by employing antibody moieties to specifically target drug delivery vehicles to blood vessels^53^^54^. found that nanoparticles significantly induce non-specific (myeloperoxidase, respiratory burst activity, etc.) and specific immune response parameters after vaccination in mice with elevated MPO. Also^55^, found that MPO levels were stimulated after vaccination with a nano-carrier. According to previous studies, nano-vaccine improves immunity and reduces inflammation, which causes cell damage^56^. Lipid peroxidase (LPO) is a biomarker for oxidative cell membrane lipid damage and one of the most widely used in vivo biomarkers for oxidative stress studies^57^. The increase in LPO level in the infected group may indicate an increase in toxic components that cause cell damage by free radicals^58^. Conversely, when bacterial infections produce oxidative stress in fish, the protective antioxidant system either weakens or increases the formation of reactive oxygen species (ROS)^59^, which includes both enzymatic and non-enzymatic antioxidants^60^. In contrast, lower value in LPO in Se-NV and Se-NV + Inf groups, which results in enhanced fish immunity and cell protection, while the Se-NV preserves the equilibrium between oxidants and antioxidants because of antioxidant enhancement or a decrease in reactive oxygen species (ROS). Additionally, the Se-NV promotes the development of immunological memory in the body by boosting the formation of cell-mediated immunity, which is generated by T and B cells and functions to destroy the pathogen or antigen^61^, as T cells excrete more thioredoxin-1 (an antioxidant) that is more resistant to ROS-induced damage (Matsushita et al., 2015). Besides that, the presence of Se-NPs, which is an effective center for Glutathione peroxidase (GPx), prevents the accumulation of free radical species and reduces cellular damage^62^.

Several anti-oxidative defence systems are used by the biological system, including enzymes such as catalase, superoxide dismutase, glutathione-S-transferase, and peroxidase, which lead to increased immunity^63^.The increased SOD and CAT values in the Se-NV and Se-NV + Inf groups could be an adaptive mechanism to immunization that neutralizes the effect of ROS and may be critical in avoiding membrane lipid peroxidation^64^. Furthermore, an increase in the CAT value may indicate an increase in the ability to remove, neutralize, or scavenge hydrogen peroxide generated in the fish body of vaccinated tilapia in response to oxidative stress caused by Se-NV injection, and this agrees with^65^. Furthermore, the presence of Se-NPs as nano carriers, which have immune-stimulatory characteristics and can increase the activity of CAT, SOD, and other antioxidants^66^. Some other anti-oxidative agents are glutathione (GSH) and glutathione-S-transferase (GST), which play an important role in modulating metal-induced LPO by acting as a reducing substrate in oxidative reactions and sequestering metals^67^. Antioxidants in GSH and GST levels in Se-NV and Se-NV + Inf groups could be an adaptive response to oxidative stress ROS (H_2_O_2_), causing an increase in antioxidant enzymes as a defense mechanism^68^. stated that H_2_O_2_ is regarded as a substrate for the GST and GSH enzymatic activities. Also, Se-NV affects macrophage activity and phagocytosis, and thus respiratory burst activity, resulting in an increase in antioxidant oxidation level in phagocytes, which is a key indicator of innate immune response^65^. Elevated GPx and GR values in Se-NV and Se-NV + Inf groups may indicate increased cellular antioxidant protection and metabolic pathway adjustment processes, in addition to reduced cytotoxicity and oxidative stress^65^. Increased GPX and GR action is very similar to the tendency noted in the case of oxidative stress induced by Se-NV injection, where H_2_O_2_ release in this oxidative stress leads to activation of GPx and GR enzymes to defend against it^67^. Aside from their role in increasing production and RBC membrane stability, nano-carriers also stimulate antioxidant enzymes such as GPx and GR. Furthermore, this agrees with the findings reported by^64^.

The *S. pyogenes* infection in the Nile tilapia results in massive hemocyte accumulations in the liver, which may be induced by free radicals because of stress^69^.The presence of numerous hemocyte accumulations in the hepatic demonstrated that the hepatic may be a target organ for hemocyte aggregation and bacterial pathogenesis^70^. These changes may result in a disorder of lipid metabolism in the hepatic tissue, known as lipidosis, which may be associated with toxins and extracellular products produced by S. pyogenes, such as Streptolysin S, and Streptolysin O^71^. Huge, diffused necrosis caused by vacuolation and atrophy in the liver of fish challenged with S. pyogenes was caused by the distribution of bacterial cells throughout the hepatic tissue^72^. The infiltration of hemocytes in hepatic tissue is a way of measuring cellular response, indicating the capabilities of the Nile tilapia to respond to the *S. pyogenes* infection. Thus, the current study’s findings showed that *S. pyogenes* can cause serious pathology in the liver of the Nile tilapia^73^. Our findings were like symptoms found in other fish species^74^. The proliferation of hematopoietic tissue and activation of the main phagocytic cells (melano-macrophages) in fish parallels hematological findings that indicate the role of bacteria and their toxins in stimulating the immune response in infected fish^75^. In contrast, the Se-NV group showed better performance with less inflammation and less hemorrhage than the infected group. For Se-NV + Inf, most of the inflammatory and oxidative stress signs were absent, with better performance, which agreed well with the hematological and biochemical results and inflammatory biomarkers. The overall lesions were high, and the inflammation was in nature.

## Conclusions

This study aimed to shed light on a new type of vaccine, which is a selenium nano-vaccine, and to determine its role on immune biochemical activity in Nile tilapia fingerlings before and after infection with *Streptococcus pyogenes*. Fish resistance was demonstrated by injecting *Streptococcus pyogenes* into the same fish that had previously received an injection of the nano-vaccine. The immunization, according to the results, improved immune response and significantly boosted bacterial resistance by increasing antioxidant enzymes and reducing oxidative stress and inflammation. Therefore, it is advised to employ nano-vaccination to strengthen fish defenses against bacterial action, and the injection approach is the preferred method for accurately dosing the fish.

## Data Availability

The datasets of the current study are available from the corresponding author.
